# Efficacy of different concentrations of lidocaine and association of vasoconstrictor in local infiltration anesthesia in adults^☆^^☆☆^

**DOI:** 10.1016/j.abd.2020.08.021

**Published:** 2021-07-16

**Authors:** Lury Bueno Wako Kitahara, Vanessa Paula da Silva, Gabriel Peres, Hélio Amante Miot, Juliano Vilaverde Schmitt

**Affiliations:** Faculty of Medicine, Universidade Estadual Paulista, Botucatu, SP, Brazil

Dear Editor,

Local infiltration anesthesia blocks nerve conduction without causing central nervous system depression. In dermatological surgeries, lidocaine is the most widely used anesthetic worldwide; however, its toxicity is dose-dependent, limiting the use of higher volumes in larger surgeries.^1^, ^2^

In Brazil, the lidocaine concentration predominantly used in dermatological procedures is 2%, with the association of vasoconstrictors, such as epinephrine, aiming to reduce intraoperative bleeding, prolong the anesthetic effect, in addition to delaying its systemic absorption, allowing the use of higher volumes, with a lesser risk of toxicity.^2^

Cutaneous infiltration of these solutions is associated with some discomfort. Thus, it is important to study strategies to minimize the patient’s pain during anesthesia, favoring their collaboration, promoting greater anesthetic success, in addition to humanizing the procedure.^3^

Warming up the anesthetic solution, slow and subcutaneous infiltration, alkalinization of the solution, previous use of a topical anesthetic, thinner needles, hypnotherapy, pre-anesthetic medications, skin vibration, and cooling, are some well-described strategies aimed at minimizing the pain associated with local infiltration anesthesia.^2^, ^3^, ^4^, ^5^ However, there have been few systematic studies reporting on the concentrations of lidocaine and epinephrine related to the painful sensitivity of the infiltration and its effectiveness in dermatological procedures.^6^

The aim of this study was to evaluate lidocaine concentrations and their association with vasoconstrictors related to the duration of anesthesia and pain due to intradermal infiltration in the forearm of adult patients.

For this purpose, a double-blind experimental model was used, involving ten adult volunteers. The project was approved by the institution's Research Ethics Committee (n. 2,647,476), and all participants signed the informed consent form. Adult patients (aged >18 years) were included, without forearm dermatoses, hypersensitivity to lidocaine, acuphobia, or coagulation disorders.

After antisepsis, the following solutions were injected slowly intradermally by the same dermatologist: 0.1 mL of a 0.5%, 1.0%, or 2% lidocaine solution with and without epinephrine (1: 200,000), and NaCl 0.9% (control), in a blinded and radnomized design, on the ventral surface of the forearm. Insulin syringes (BD-ultrafine) were used. All ten participants received all seven treatments, in a randomized sequence.

Pain intensity related to the infiltration of each solution was assessed using a visual analog scale (VAS: 0–10 points). Subsequently, the presence of pain was evaluated at the standardized puncture with lancets used in the prick test for capillary blood glucose (5 mm), using a specific pen, at times zero, 15, 30, 45, and 60 min, over the infiltrated areas. After 60 min, a new sensitivity assessment was carried out after transdermal perforation with a 30G needle.

The study dependent variables were the pain score at the infiltration and the presence of painful sensitivity, compared for each of the seven treatments, in a dependent manner, according to the ten participants and the evaluation time. The infiltration pain scores and the presence of painful sensitivity at different times were compared regarding the effect of lidocaine concentration, use of epinephrine and the drug interaction through generalized linear models of mixed-effects (normal distribution and logit), with an unstructured covariance matrix, and Sidak’s post hoc test. A p-value of ≤0.05 was considered significant. The sample size was based on the expectation of a difference >30% in the proportion of pain by any of the compositions, with an alpha error = 0.05 and beta = 0.2.

Of the study volunteers, 5 (50%) were females, with ages ranging from 20 to 43 years. The pain scores resulting from the intradermal infiltration of each solution are shown in Fig. 1. There was a slight increase in the mean infiltration pain score in the participants who received epinephrine, compared to those who did not receive it (5.8 [sd = 2.1] vs 5.0 [sd = 2.2]; p = 0.013). Similarly, higher infiltration pain scores were observed in participants who received high concentrations of lidocaine (2%), when compared to those receiving lower concentrations (1% and 0.5%) concentrations (6.6 [sd = 1.7] vs. 5.1 [sd = 2.1] and 4.5 [sd = 1.8]; p < 0.001).

**Figure 1:**
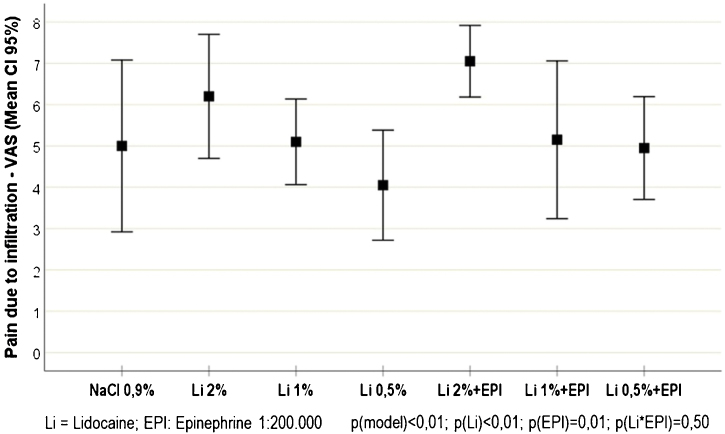
Pain intensity (VAS - Visual Analogue Scale: 0–10) reported at the time of the injections of the different tested solutions (n = 70).

The frequencies of painful sensitivity after the standardized stimulus for each anesthetic solution, are shown in Fig. 2. There was no painful sensitivity with the 2% lidocaine solution with epinephrine throughout the experiment, while the other solutions maintained anesthesia for up to 30 min in all cases. The painful sensitivity progressed in relation to time and was inversely proportional to the lidocaine concentration (p < 0.001); similarly, the anesthesia lasted longer when epinephrine was used (p = 0.046). The post hoc analysis showed that, between 0 and 45 min, there was no difference in the anesthetic profile for 2% and 0.5% lidocaine (p > 0.15).

**Figure 2:**
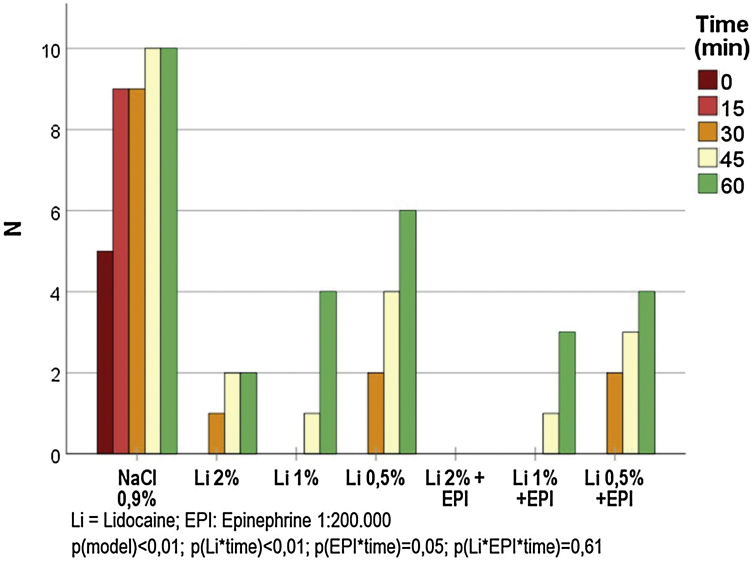
Frequency of painful sensation (standardized stimulus) in relation to time, after injections of the different tested solutions (n = 350).

Fig. 3 shows the frequency of sensitivity to an intense painful stimulus after 60 min of the infiltration of each solution. None of the participants had pain in the region where 2% lidocaine was infiltrated with epinephrine. A lower frequency of sensitivity was observed in the solutions that used epinephrine (30% vs. 70%; p < 0.001), as well as, proportionally, in the solutions that used the highest concentrations of lidocaine (0.5%; 1% and 2%): 38% vs. 27% vs. 8% (p < 0.001). A positive interaction between epinephrine and lidocaine was identified at a concentration of 1% (40% vs. 80%; p = 0.035), whereas a negligible significance was observed for the interaction with 2% lidocaine (0% vs. 60%; p = 0.070).

**Figure 3:**
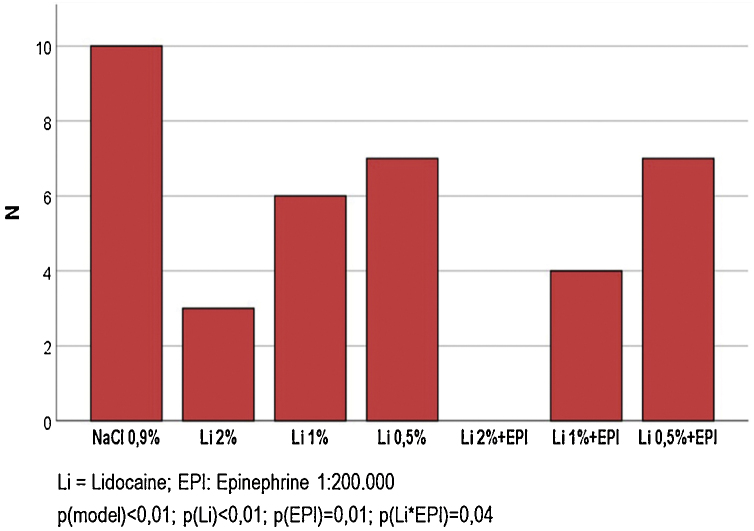
Frequency of painful sensation (most intense stimulus) 60 minutes after the injections of each of the different tested solutions (n = 70).

Pain related to the process of local anesthetic infiltration is associated with elements related to the patient, the application technique and the used solutions.^3^ Our results showed different behaviors regarding the effectiveness and pain of infiltration when the concentrations of lidocaine and associated epinephrine varied.

The association of epinephrine and lidocaine ratified its effect by extending the duration of anesthetic effectiveness.^2^ The increase of pain in the infiltration associated with epinephrine can be compensated, for instance, with lower concentrations of lidocaine, or alkalinization techniques, such as the addition of bicarbonate, without impairing the anesthetic effect.^7^, ^8^

Lidocaine at concentrations lower than 2% also showed no difference in the anesthetic profile of the first 30 min, confirming the results from the tumescent anesthesia technique.^1^, ^9^ Lower concentrations of lidocaine, when associated with epinephrine, increase the safety of anesthesia, especially when larger volumes of anesthetics are needed.^6^, ^10^

The study has some limitations associated with its experimental characteristic, which does not consider the anxiety that is naturally involved in dermatological surgeries, in addition to the reduced volume of anesthetic used. In contrast, the intradermal injection promotes a faster effect, with more pain at the injection. Finally, the use of only one topography hampers the generalization of the results to other areas with greater sensory innervation, such as the face. All of these elements have been standardized aiming to maximize the comparability of individuals.

In conclusion, lidocaine, at concentrations of 0.5% or 1%, promotes less pain at intradermal infiltration without anesthetic impairment in the first 30 min, and epinephrine increases the effectiveness and duration of local anesthesia.

## Financial support

None declared.

## Authors’ contributions

Lury Bueno Wako Kitahara: Data collection; writing of the text; literature review; final approval of the manuscript.

Vanessa Paula da Silva: Data collection; writing of the text; literature review; final approval of the manuscript.

Gabriel Peres: Data collection; project concept; data analysis; writing of the text; literature review; final approval of the manuscript.

Hélio Amante Miot: Data collection; project concept; data analysis; writing of the text; literature review; final approval of the manuscript.

Juliano Vilaverde Schmitt: Data collection; project concept; data analysis; writing of the text; literature review; final approval of the manuscript.

## Conflicts of interest

None declared.
